# Identifying “at-risk” patients for sub-optimal beta-lactam exposure in critically ill patients with severe infections

**DOI:** 10.1186/s13054-017-1871-2

**Published:** 2017-11-21

**Authors:** Mohd H. Abdul-Aziz, Jeffrey Lipman, Jason A. Roberts

**Affiliations:** 1 Faculty of Medicine, University of Queensland Centre for Clinical Research (UQCCR), The University of Queensland, Building 71/918 Royal Brisbane & Women’s Hospital, Brisbane, QLD 4029 Australia; 2 School of Pharmacy, International Islamic University Malaysia, Kuantan, Malaysia; 3 Department of Intensive Care Medicine, Royal Brisbane and Women’s Hospital, Brisbane, QLD Australia; 4 Centre for Translational Anti-infective Pharmacodynamics, School of Pharmacy, The University of Queensland, Brisbane, QLD Australia; 5 Department of Pharmacy, Royal Brisbane and Women’s Hospital, Brisbane, QLD Australia

**Keywords:** Augmented renal clearance, Critically ill, Meropenem, Pharmacokinetic, Pharmacodynamic, Risk assessment tool

## Pathophysiological changes affecting drug pharmacokinetics

Mortality due to severe infections in the intensive care unit (ICU) remains high despite recent therapeutic advancements [1]. However, appropriate antibiotic administration (including spectrum of activity and therapeutic exposure) is rarely a straightforward process in ICU patients as they commonly develop extreme pathophysiological changes that can alter antibiotic pharmacokinetics and consequently affect drug exposure in this population. The volume of distribution and drug clearance are the pharmacokinetic parameters of greatest relevance to determining drug dosing requirements, and both parameters may be significantly deranged during critical illness [2, 3]. Furthermore, ICU pathogens are relatively different from those in the general wards as they commonly have reduced antibiotic susceptibility [4]. Despite profound physiological and pharmacokinetic differences to the non-critically ill population, critically ill patients are typically given conventional antibiotic dosing regimens, which increase the likelihood of therapeutic failures and the emergence of bacterial resistance [5].

## Optimal pharmacodynamic index for maximal beta-lactam activity

Beta-lactam antibiotics display time-dependent pharmacodynamics, whereby the time for which the free (unbound) drug concentrations remain above the minimum inhibitory concentration (*f*T_> MIC_) best characterises bacterial killing [6]. Specifically, the %*f*T_> MIC_ value needed for bactericidal activity is between 40 and 70% for these antibiotics. However, emerging clinical data from critically ill patients suggest that these patients may benefit from higher and longer antibiotic exposures [7, 8]. It has since been suggested that beta-lactam concentrations should be maintained as at least four or five times the MIC for extended periods during each dosing interval (e.g. 90–100% T_> 4×MIC–5×MIC_) to maximise patient outcomes, including suppressing the emergence of resistant bacteria [9]. Nevertheless, achieving this aggressive pharmacokinetic/pharmacodynamic target is not easy in critically ill patients, particularly when standard beta-lactam dosing is applied.

## Pharmacokinetic/pharmacodynamic derangements during critical illness

The DALI study has reported significant variability in beta-lactam exposures in critically ill patients [5]. In this study, whilst plasma beta-lactam concentrations could vary by up to 500-fold, pharmacokinetic/pharmacodynamic exposures varied by more than 1000-fold. Approximately one-fifth of the DALI cohort failed to achieve even the most conservative pharmacokinetic/pharmacodynamic target (50% *f*T_> MIC_) with standard beta-lactam dosing and these patients were 32% more likely to demonstrate negative outcomes (e.g. prolonged antibiotic courses).

The complexity of beta-lactam dosing in critically ill patients has been characterised recently by Ehmann et al. [10] in their prospective, observational, single-centre pharmacokinetic study. In this study, 48 critically ill patients with severe sepsis were recruited, with the investigators evaluating the pharmacokinetic/pharmacodynamic target attainment of standard meropenem dosing (1000/2000 mg every 8 hours as a 30-min infusion) in critically ill patients. The investigators then developed a tool that may improve meropenem exposure in this population. Large variation in meropenem concentration was observed in this study, corroborating the findings of earlier studies [2, 5, 11, 12]. The pharmacokinetic/pharmacodynamic target attainment of the cohort was relatively poor; only 56% and 48% of the cohort achieved the tested pharmacokinetic/pharmacodynamic targets of 50% *f*T_> 4×MIC_ and 100% *f*T_> MIC_, respectively, against the MIC susceptibility breakpoint of 2 mg/L. It is also noteworthy that the investigators had chosen to use two aggressive pharmacokinetic/pharmacodynamic targets as opposed to the conventional target for optimal beta-lactam activity (50% *f*T_> MIC_). Furthermore, the MIC values were assumed from population estimates (EUCAST MIC breakpoints), inflating the magnitudes of target non-attainment in their analysis.

## Sub-therapeutic beta-lactam exposure and the influence of renal function

As the beta-lactams are predominantly cleared by the kidney, elevated renal function may likely lead to sub-optimal antibiotic exposure, particularly when conventional dosing regimens are used [2, 13]. Patients with severe infections commonly develop a systemic inflammatory response syndrome, which increases renal blood flow and glomerular filtration rates. These factors enhance renal clearance of some drugs, a phenomenon referred to as augmented renal clearance (ARC). A measured creatinine clearance (CL_CR_) ≥ 130 ml/min has been used to correlate ARC with sub-optimal antibiotic exposures [13]. Although ARC is highly prevalent in most ICUs [14], most clinicians fail to address the phenomenon, persisting with conventional beta-lactam dosing that is likely flawed, particularly when less susceptible pathogens are present.

In their study, Ehmann et al. [10] observed that increasing estimated CL_CR_ significantly reduced the likelihood of pharmacokinetic/pharmacodynamic target attainment. This further highlights that those patients who are at risk for ARC, usually those with apparently “normal” renal function, have to be identified earlier so that dose modification can be made earlier [2, 13]. In this respect, the investigators are certainly heading in the right direction with their proposed solution. They have developed a practical tool in commonly used software (Microsoft Excel) to predict the risk of target non-attainment for non-RRT critically ill patients. This free and easy-to-use risk assessment tool, the MeroRisk Calculator, would be able to predict the likelihood of achieving 100% T_> MIC_ with standard meropenem dosing by inputting the CL_CR_ of a patient or its determinants together with the MIC of a known or suspected pathogen. The calculated risk of target non-attainment is displayed with a three-colour coding system and at-risk patients are highlighted in red, which should prompt clinicians to alter dosing for such patients. Although the MeroRisk Calculator was developed based on a broad range of CL_CR_ (25–255 ml/min), the prediction uncertainty increases for the extremes of renal function due to the limited number of patients representing this sub-population. The tool also provides a graphical illustration of the relationship between estimated CL_CR_ and the predicted meropenem exposure which therefore describes the degree of uncertainty around their prediction.

This promising tool, however, can be improved upon. It was developed based on the Cockcroft–Gault CL_CR_, but the measured CL_CR_ is likely to be more appropriate in the ICU, particularly in patients with ARC [15]. Severity of illness may influence meropenem exposure, particularly in terms of the volume of distribution, and its impact should be incorporated into the Calculator. Actual MIC must be provided for accurate prediction as opposed to population estimates. The MeroRisk Calculator should be refined to also include other patient sub-groups, namely ECMO and RRT patients, in the prediction model.

## Conclusion

Conventional beta-lactam dosing is flawed in critically ill patients. Useful tools such as the MeroRisk Calculator need to be comprehensively evaluated clinically, and if successful should be added into clinical practice to guide effective antibiotic dosing.

## Abbreviations

ARC: Augmented renal clearance
CL_CR_: Creatinine clearance
DALI: Defining Antibiotic Levels in Intensive care unit patients
ECMO: Extracorporeal membrane oxygenation
ICU: Intensive care unit
MIC: Minimum inhibitory concentration
RRT: Renal replacement therapy
T_> MIC_: Time for which drug concentration remains above the minimum inhibitory concentration during a dosing interval
